# Supplementary material for: Climatic niche comparison between closely related trans-Palearctic species of the genus Orthocephalus (Insecta: Heteroptera: Miridae: Orthotylinae)
Source: PeerJ. 2020 Dec 15;8:e10517. doi: 10.7717/peerj.10517 (PMC7747689; doi:10.7717/peerj.10517)
Supplement: Supplemental Information 4 [file peerj-08-10517-s004.docx]

***Descriptions of the climatic models and variables ranges for each species***

*Orthocephalus bivittatus*

**Geographic distribution**. Most specimens used in this analysis were collected in South Russia, Caucasus and Central Asian countries. The most western record is from Hungary, the most eastern record is from Eastern Siberia near the lake Baikal, the most northern record is from central European Russia (Bryansk Province), and the most southern is from the southern Turkmenistan near the boarders with Iran and Afghanistan (Fig. 1A).

In both models suitable conditions mainly extend from Eastern Europe (12-13°E) to Western China and Mongolia (95-96°E) in longitudinal direction, and from the middle of European Russia (30-31°N) to Iran and Afghanistan (58-59°N) in the latitudinal direction. Only very small patches of suitable conditions appear beyond those limits. However, the CF model predicts large areas of suitable conditions in eastern and northern Europe and central and northern European Russia up to northern Scandinavia and Karelia, whereas only small patches of suitable conditions are predicted there by the CR model. Both models do not predict Middle and Western Europe to be very suitable for *O. bivittatus* (Figs 3A, 4A).

***Climatic variables***. Both models show that the annual average temperature (bio1) significantly contributes to the distribution, having both PC and PI high. In respect to other variables, the models are very different. In the CF model mean temperature of coldest quarter (bio11) is important too, and this model is also explained by the variables, connected with the annual, seasonal and diurnal temperature changes (bio2-4, bio7), as well as precipitation seasonality (bio15). In the CR model among the temperature variables, mean temperatures of driest quarter and month (bio9, bio10) are important, as well as precipitation seasonality (bio15) and precipitation of warmest quarter (bio18) (Table 2). Among all those variables, bio1 and bio11 (PCor = 0.93) and bio4 and bio7 (PCor=0.94) significantly correlate with each other (Supplemental Data 2).

The variable ranges show that precipitation in the driest month and driest, warmest and coldest quarters (bio14, bio17-19) have relatively restricted ranges and shifted towards less precipitation in comparison to that of most other species (Figs10, 11). Suitable conditions for *O. bivitattus* are not modelled for the places with low max temperature of warmest month (bio5) and annual temperature range (bio7), as the lowest values of these variables are higher than in other species (Fig. 8).

*Orthocephalus brevis*

**Geographic distribution**. Most records used in the current analysis are from Middle and Eastern Europe, western Russia, Caucasus and western Siberia (Fig 1B).

Both models predict a large area of suitable conditions in Western Palearctic from Portugal (8°W) to Ural Mountains (55-56°E) in longitudinal direction, and from north of Spain, Italy, Greece, Tukey and Iran (37-38°N) to northern Scandinavia (65-66°N) in the latitudinal direction. Additionally, Northeast Asia is predicted as suitable for *O. brevis*. There are also small patches of suitable conditions in Altai Mountains and Central Asia up to India (Figs 3B, 4B).

**Climatic variables**. Both models show that the annual mean temperature (bio1) and precipitation of driest month (bio14) significantly contribute to the distribution with high PC and PI in both cases. In the CR model precipitation seasonality (bio15) has high PI. None of those variables significantly correlate with each other.

In comparison with other species, precipitations of driest month, driest and coldest quarters (bio14, bio17, bio19) in both models are high, and never reach 0 (Figs 10, 11). The lowest limit of annual precipitation (bio12) is also higher than in most of other species (Fig. 9) and the upper limits of diurnal range (bio2) is low in comparison to most of the other species (Fig. 7). The precipitation ranges show that *O. brevis* generally prefers wetter places than other species.

*Orthocephalus coriaceus*

**Geographic distribution**. In this analysis the records from Central and Southern Europe, as well as from Kyrgyzstan and Tunisia, are used (Fig. 1C).

The overall size of areas with suitable conditions is distinctly smaller in the CR model than in the CF model, i.e. 5.08 x10^6^ km^2^ and 8.19 x10^6^ km^2^ respectively (Table 2). The models predict suitable conditions in most of Europe and partly in Near East, up to Portugal in the west (8°W) up to Northern Iran in the east (54-55°E). However, the CR model mostly does not cover the areas in Belarus, Ukraine and western Russia, whereas in the CF model suitable conditions extend up to the middle parts of the European Russia. Both models estimate suitable conditions from northern Africa and northern Iran (34-36°N) up to Scandinavia in latitudinal direction. The CR model is more restrictive and estimates suitable conditions up to southern Scandinavia (61-62°N), whereas in the CF model they are extended more to the north (67°N). Both models predict suitable conditions in Iceland. Only small patches of suitable conditions are predicted beyond those areas (Figs 3C, 4C).

**Climatic variables**. Both models show that isothermality, precipitation of driest month and coldest quarter (bio3, bio14, bio 19) significantly contribute to the distribution with high PC and PI. Among other variables, mean temperate of coldest quarter (bio11) and precipitation seasonality (bio15) are important for the CF model, and precipitation of wettest quarter (bio16) is important for the CR model (Table 2). Among them bio19 significantly correlates with bio3 and bio14 (PCor = 0.71, 0.76 respectively), bio14 additionally correlates with bio15 and bio16 (PCor = -0.85, 0.79 respectively), and bio3 correlates with bio11 (PCor = 0.84) (Supplemental Data 2).

Variable ranges for isothermality (bio3) have lowest limits with higher values than in other species and relatively small range in comparisons to most of other species (Fig. 7). The models predict the lowest margins of mean temperature of warmest quarter (bio10) to be lower than in many other species, almost reaching 0 (Fig. 9), as the model predicts suitable conditions for *O. coriaceus* in northern Scandindavia and Iceland.

*Orthocephalus fulvipes*

**Geographic distribution**. In this analysis only records from northern Africa and Near East countries are used (Fig. 1D).

Both models are very similar and predict suitable conditions from Portugal and Morocco in the west (8-9°W) to Tadjikistan and Kyrgyzstan in the east (70-71°E), from the middle of Algeria and south of Arabian Peninsula (20-24°N) in the south to south Kazakhstan, north of Turkey as well as the Northern Mediterranean (39-42°N) in the north. Only small patches of suitable conditions are predicted beyond this area (Figs 3D, 4D).

**Climatic variables**. Precipitation of warmest quarter (bio18) is important for both models. In the CF model mean temperature of driest quarter (bio9) and precipitation of coldest quarter (bio19) are significantly important. In the CR model mean temperature of coldest month (bio6) and precipitation of warmest quarter (bio16) are additionally important (Table 2). Among those variables only bio16 and bio19 strongly correlate with each other with PCor = 0.97 (Supplemental Data 2).

The ranges of many temperature related variables are shifted towards higher values, which are annual mean temperature (bio1), isothermality (bio3), max temperature of warmest month (bio5), mean temperature of coldest month, driest, warmest and coldest quarters (bio6, bio9-bio11) (Figs 7-9). Precipitation variables values are often restricted by the relatively low values, e.g. annual precipitation (bio12), precipitation of the direst month, driest and warmest quarters (bio14, bio17, bio18) (Figs 9-11). This suggests that suitable conditions cover many areas with relatively high temperatures all year and low precipitations at least over the warmest and driest periods.

*Orthocephalus funestus*

**Geographic distribution**. For the analysis localities from Russian Far East, Japan, Korea, northeastern and northern China were used, and this area covers almost the entire known distribution of *O. funestus*, except for Mongolia (Fig 2A).

Both models predict suitable conditions from southeastern China (23°N) in the south to the border between Khabarovsk Kray and Magadan Province, Russia (57-58°N) in the north. Both models cover Japan, Sakhalin Island and south of Kamchatka Peninsula. The CR model is more restricted in latitudinal directions and suitable conditions extend up to 131-135°E in Russia (north of Khabarovsk Kray) and northern China (Heilongjiang Province) and up to 110°E in southeastern China (Hubei Province), whereas the CF model predicts suitable conditions up to 124°E in Russia (west of Amur Province) and 103°E in southeastern China (Sichuan Province). Both models predict large areas of suitable conditions in the European part of Russia and small patches in other Palearctic regions (Figs 5A, 6A).

**Climatic variables**. In both models isothermality (bio3) has both PC and PI high. Annual mean temperature (bio1) and precipitation of wettest and warmest quarters (bio16, bio18) also significantly contribute to the CF model. In the CR model mean temperature of coldest month and warmest quarter (bio6, bio10), precipitation seasonality (bio15) and precipitation of driest quarter (bio17) are important (Table 2). All the temperature related variables correlate with each other with PCor > 0.7, except for bio6, which significantly correlates with bio1 only (PCor = 0.85). Bio1 and bio3 correlate with bio16 and bio18 with PCor ~0.7-0.8. Bio15 and bio17 correlate with PCor =0.77, bio16 and bio18 strongly correlate with PCor = 0.99 (Supplemental Data 2).

The annual mean temperature (bio1), min temperature of coldest month (bio6), mean temperature of driest and coldest quarters (bio9, bio11) have lower margin shifted towards lower values in comparison to other species (Figs 7-9). Isothermality (bio3) is limited with the lower values in comparison to other species, whereas temperature seasonality (bio4) and temperature annual range (bio7) are shifted towards higher values than in many other species (Figs 7, 8). Many ranges of precipitation related variables are shifted towards higher values or have upper margins limited with the higher values in comparison to many other species, i.e. annual precipitation (bio12), precipitations of wettest and driest months, wettest, driest and warmest quarters (bio13, 14, 16, 17, 18) (Figs 9-11). Those ranges suggest that *O. funestus* has suitable areas in places with strong seasonality, the temperature in coldest and driest periods lower than in other species, and relatively high precipitation in all periods.

*Orthocephalus proserpinae*

**Geographic distribution**. In this analysis the localities from south European countries and North Africa (Fig. 2B) are used. All models estimate suitable conditions around the Mediterranean Sea from Morocco and Portugal (9-13°W) in the west to Syria and Jordan (36-37°W) in the east, from Morocco or Western Sahara (22-27°N) in the south to southern France, northern Italy, Greece and Turkey in the north (40-42°N) (Figs 5B, 6B).

**Climatic variables**. In the CF and CR models temperature seasonality (bio4), precipitation of driest month and coldest quarter (bio14, bio19) are also important. Temperature annual range (bio7) and mean temperature of driest quarter (bio9) significantly contribute to the CF model (Table 2). None of those variables significantly correlate with each other.

Isothermality (bio3) range is shifted towards higher ranges, whereas temperature seasonality (bio4) and temperature annual range (bio7) are limited by the relatively low values (Figs 7, 8). The values of min temperature of coldest month (bio6), mean temperature of driest and coldest quarters (bio9, bio11) are confined by the relatively high values (Figs 8, 9), whereas precipitation of driest month and driest quarters (bio14, bio17) are confined to relatively low values (Figs 10, 11). Those ranges show that *O. proserpinae* prefers places with low temperature changes over the year, high temperatures of the coldest and driest periods, and low precipitation over the driest season.

*Orthocephalus saltator*

**Geographic distribution**. In this analysis numerous localities from around Europe, European and Asian Russia, Caucasus, Turkey, Iran, Kazakhstan are used (Fig. 2C). All models cover most of the Western Palearctic from Morocco and Spain (8-9°W) in the west to Volga River and northern Iran in the east (52-55°E), from northern Africa (30-31°N) in the south to northern Sweden and White Sea (64-65°N) in the north. Both models also predict large areas of suitable conditions in southern Urals and Altai Mountains. The small patches of suitable conditions also occur in Central Asia and Norheast Asia (Figs 5C, 6C).

**Climatic variables**. The models are mostly explained by the temperature related variables (Table 2). Min temperature of coldest month (bio6) is important for both models, and mean annual temperature (bio1) is important for the CF models, those two variables strongly correlate with each other (PCor = 0.97). The CF model is also explained by the temperature annual range (bio7) and mean temperature of coldest quarter (bio11), which also strongly correlate with each other, as well as with bio1 and bio6 (PCor > 0.8 or PCor < -0.8). In the CF model precipitation of coldest quarter is important (bio19), which significantly correlates with bio6 and bio7 (PCor ~ 0.7-0.8).

Variable ranges for *O. saltator* do not show any noticeable peculiarities in comparison to most of the other species.

*Orthocephalus vittipennis*

**Geographic distribution**. In the current analysis the records from the Eastern and Middle Europe (Czech Republick, Poland) in the west to Yakutia in the east, from Finland and Karelia in the south to Balkans, Iran, Caucasus, and Turkmenistan in the south are used (Fig 2D).

The models predict suitable conditions from Spain to Japan. In the eastern Palearctic the models predict suitable conditions up to southern Europe, excluding most part of Mediterranean region, and northern Iran (38-39°N) in the south. In the north the models extend up to the northern Scandinavia and Kola Peninsula (65-69°N). In eastern Palearctic the models predict suitable conditions up to 62-64°N in the north, and up to Tajikistan and Kyrgyzstan (38-39°N) and south China (26-27°N) in the south (Figs 5D, 6D).

**Climatic variables**. In both models mean temperature of driest quarter (bio9) significantly contributes. Annual temperature range (bio1) and mean temperature of coldest quarter (bio11) additionally significantly contribute to the CF model. Mean diurnal range (bio2), annual temperature range (bio7), precipitation of wettest and warmest quarters (bio16, bio18) are important for the CR model (Table 2). All temperature related variables significantly or strongly correlate with each other (PCor = 0.7-1), except for bio2. Bio16 significantly correlates with bio18 (PCor = 0.76).

The variable ranges for mean annual temperature (bio1), mean temperature of coldest month (bio6), mean temperature of driest and coldest quarters (bio9, bio11) are limited by the values lower than in many other species (Figs 7-9). Isothermality (bio3) reaches the lower values in comparison with many other species, and temperature seasonality (bio4) and temperature annual range (bio7) reach the highest values among all species (Figs 7, 8). The upper margin of the precipitation of coldest quarter (bio19) is limited by the relatively low values (Fig. 11). Harsh conditions with extreme seasons and very low temperatures over winter can be suitable for *O. vittipennis*.
