# Supplementary material for: Climatic niche comparison between closely related trans-Palearctic species of the genus Orthocephalus (Insecta: Heteroptera: Miridae: Orthotylinae)
Source: PeerJ. 2020 Dec 15;8:e10517. doi: 10.7717/peerj.10517 (PMC7747689; doi:10.7717/peerj.10517)
Supplement: Supplemental Information 5 [file peerj-08-10517-s005.docx]

Supplementary data S2. All sets of parameters and variables, used for the niche modelling, with corresponding AUC values and omission rates. Omission rate corresponds to “10 percentile training presence test omission” in Maxent results. The sets of parameters and variables used for the visualization and niche comparison are in bold.

| species | parameters | variables | AUC (Training) | AUC (Test) | Omission rate |
| --- | --- | --- | --- | --- | --- |
| **bivittatus** | **LQ1.5** | **all** | **0.928** | **0.926** | **0.0927** |
| bivittatus | L3.5 | 1-3, 8-10, 13-15, 18-19 | 0.836 | 0.812 | 0.1146 |
| **bivittatus** | **LQ1** | **1, 3, 4, 8-10, 13-15, 18, 19** | **0.925** | **0.929** | **0.0829** |
| bivittatus | LQH2.5 | 1-4, 8-10, 12, 15, 17-19 | 0.947 | 0.934 | 0.1415 |
| bivittatus | H2 | 2, 3, 8-10, 12, 15, 17-19 | 0.944 | 0.94 | 0.1146 |
| bivittatus | LQHP3.5 | 2-4, 8-12, 15, 17-19 | 0.947 | 0.944 | 0.1122 |
| bivittatus | LQHPT3.5 | 2-4, 8-12, 14, 15, 18, 19 | 0.953 | 0.943 | 0.1415 |
| brevis | H2 | all | 0.942 | 0.918 | 0.1471 |
| **brevis** | **H5.5** | **all** | **0.921** | **0.911** | **0.1206** |
| brevis | H1.5 | all | 0.941 | 0.928 | 0.15 |
| brevis | L6 | 1, 8, 10, 15-18 | 0.844 | 0.829 | 0.1294 |
| brevis | LQ6 | 8-10, 15-17 | 0.875 | 0.856 | 0.13 |
| brevis | LQH1.5 | 1-3, 7-10, 12, 14-16, 18, 19 | 0.948 | 0.93 | 0.1559 |
| brevis | H6 | 5, 8, 11, 12, 14, 15 | 0.907 | 0.901 | 0.1 |
| **brevis** | **LQHP6** | **1, 5, 9, 12-15, 18** | **0.917** | **0.912** | **0.1** |
| brevis | LQHPT6 | 10-12, 14-16 | 0.918 | 0.9 | 0.1177 |
| **coriaceus** | **LQH2** | **all** | **0.966** | **0.947** | **0.1176** |
| coriaceus | L1.5 | 3, 4, 10, 16, 18 | 0.854 | 0.85 | 0.1471 |
| coriaceus | LQ1 | 1, 3, 4, 10, 11, 13, 18, 19 | 0.941 | 0.929 | 0.1765 |
| coriaceus | LQH2 | 1-3, 11, 16, 18, 19 | 0.964 | 0.949 | 0.2059 |
| coriaceus | H1.5 | 1-3, 14, 16, 18, 19 | 0.966 | 0.942 | 0.1471 |
| **coriaceus** | **LQHP1.5** | **2, 3, 14, 16, 18, 19** | **0.969** | **0.953** | **0.1125** |
| coriaceus | LQHPT2 | 2, 3, 7, 14, 16, 18 | 0.972 | 0.961 | 0.15 |
| fulvipes | LQH2.5 | all | 0.98 | 0.973 | 0.1111 |
| **fulvipes** | **LQH3** | **all** | **0.980** | **0.975** | **0.1111** |
| fulvipes | L1 | 4, 6, 14, 19 | 0.948 | 0.935 | 0.1667 |
| fulvipes | LQ0.5 | 5, 7, 8, 11, 14, 16 | 0.973 | 0.966 | 0.1667 |
| fulvipes | LQH2.5 | 5, 6, 14, 16 | 0.977 | 0.962 | 0.2222 |
| fulvipes | H4 | 5, 19 | 0.947 | 0.945 | 0.1111 |
| fulvipes | LQHP3.5 | 5, 14, 19 | 0.976 | 0.966 | 0.2222 |
| **fulvipes** | **LQHPT3.5** | **5, 6, 16, 18** | **0.983** | **0.98** | **0.1111** |
| funestus | LQ4 | all | 0.975 | 0.974 | 0.1158 |
| funestus | LQ3.5 | all | 0.977 | 0.968 | 0.1421 |
| **funestus** | **H4.5** | **all** | **0.976** | **0.973** | **0.1053** |
| funestus | H4 | all | 0.973 | 0.969 | 0.1263 |
| funestus | L4 | 1-3, 7, 8, 14 | 0.906 | 0.901 | 0.1211 |
| funestus | LQ3.5 | 2, 3, 7- 9, 14 | 0.921 | 0.907 | 0.1263 |
| funestus | LQH5 | 2, 3, 7, 9, 14 | 0.845 | 0.831 | 0.1211 |
| **funestus** | **H2.5** | **2, 3, 6, 10, 15, 17** | **0.979** | **0.976** | **0.0789** |
| funestus | LQHP5.5 | 2, 3, 6, 12, 15, 18, 19 | 0.968 | 0.967 | 0.0790 |
| funestus | LQHPT5 | 2, 3, 6, 12, 18, 19 | 0.968 | 0.965 | 0.948 |
| **proserpinae** | **LQ4** | **all** | **0.993** | **0.988** | **0.1111** |
| proserpinae | LQH4 | all | 0.993 | 0.989 | 0.2222 |
| proserpinae | L1 | 4, 6, 7, 14, 15 | 0.961 | 0.957 | 0.1667 |
| proserpinae | LQ0.5 | 3, 4, 6, 7, 14, 15, 19 | 0.991 | 0.988 | 0.1111 |
| proserpinae | LQH2.5 | 2, 4, 14, 15, 19 | 0.993 | 0.989 | 0.1667 |
| proserpinae | H1 | 2, 4, 15, 16, 18 | 0.994 | 0.990 | 0.1111 |
| proserpinae | LQHP2.5 | 2, 4, 14, 15, 19 | 0.993 | 0.990 | 0.1667 |
| **proserpinae** | **LQHPT3** | **4, 7, 14, 15, 19** | **0.993** | **0.991** | **0.1111** |
| saltator | H4 | all | 0.919 | 0.9 | 0.1304 |
| **saltator** | **LQ0.5** | **all** | **0.915** | **0.908** | **0.1125** |
| saltator | L6 | 3, 7, 8, 9, 13, 15, 17, 18, 19 | 0.801 | 0.797 | 0.0982 |
| saltator | LQ0.5 | 2-6, 8, 9, 12-15, 18, 19 | 0.915 | 0.908 | 0.1232 |
| saltator | LQH3 | 2-4, 6, 8, 9, 13-15, 18, 19 | 0.921 | 0.901 | 0.1286 |
| saltator | H1.5 | 1-3, 7-10, 12-15, 18, 19 | 0.930 | 0.918 | 0.1232 |
| **saltator** | **LQHP2.5** | **2-6, 8, 9, 12-15, 18, 19** | **0.93** | **0.925** | **0.1214** |
| saltator | LQHPT2.5 | 2, 3, 5-9, 12-15, 18, 19 | 0.937 | 0.926 | 0.1268 |
| **vittipennis** | **LQ0.5** | **all** | **0.876** | **0.851** | **0.162** |
| vittipennis | L4.5 | 2, 3, 7-10, 13-15 | 0.653 | 0.631 | 0.1320 |
| vittipennis | LQ0.5 | 1-4, 8-10, 12-15, 18, 19 | 0.851 | 0.828 | 0.142 |
| vittipennis | LQH1.5 | 1-3, 7-10, 14-16, 18, 19 | 0.878 | 0.845 | 0.160 |
| vittipennis | H1 | 1-3, 7, 10, 14-19 | 0.878 | 0.845 | 0.196 |
| **vittipennis** | **LQHP1.5** | **1-3, 7-10, 14-16, 18, 19** | **0.89** | **0.863** | **0.144** |
| vittipennis | LQHPT1.5 | 1-3, 7-10, 14-19 | 0.907 | 0.866 | 0.2160 |
